# Knowledge of Hemoglobin A1c and Glycemic Control in an Urban Population

**DOI:** 10.7759/cureus.13995

**Published:** 2021-03-19

**Authors:** Raafia Memon, David Levitt, Silvia R Salgado Nunez del Prado, Kashif Munir, Elizabeth Lamos

**Affiliations:** 1 Internal Medicine/Division of Endocrinology, Diabetes and Nutrition, University of Maryland School of Medicine, Baltimore, USA; 2 Internal Medicine/Endocrinology, ChristianaCare, Newark, USA; 3 Division of Endocrinology, Diabetes and Metabolism, Virginia Commonwealth University, Richmond, USA

**Keywords:** diabetes, hba1c, hemoglobin a1c, glycemic control, patient perception

## Abstract

Aim: Our study aims to assess the knowledge of hemoglobin A1c (HbA1c) and glycemic control in patients with diabetes mellitus (DM) at an urban academic institution.

Methods: This was a retrospective cross-sectional study that included a survey of 100 adult patients with DM. Our patient cohort was divided into those with recent HbA1c < 8.0% and those with HbA1c ≥ 8.0% for subgroup analysis.

Results: The majority (71%) of patients correctly defined HbA1c and half were aware of their HbA1c target, but they were unable to correlate the correct average blood glucose for an HbA1c level of 7%. Worse control, defined as an HbA1c level of ≥ 8%, was associated with co-morbid disease, but was not associated with understanding HbA1c definition, target or socioeconomic disparities. Perceived glycemic control was congruent with the actual control in 46% of our patients. Ninety percent of those with HbA1c ≥ 8% perceived their control to be better than it actually was, and 97% of those with HbA1c < 8% perceived their control worse than it actually was (P < 0.00001).

Conclusion: Although most patients knew the definition of HbA1c, they were unable to correlate HbA1c with average blood sugar. There remain opportunities to increase education for this vulnerable population with co-morbid disease on the use of the HbA1c disease marker as an education tool.

## Introduction

The lack of understanding of patients’ role in their medical care and knowledge of their chronic disease condition and therapeutic goals can often be a barrier to optimal control. Evidence suggests that patients with chronic disease, who are engaged and active participants in their medical care, have better health outcomes [1]. The hemoglobin A1c (HbA1c) test, an integral component of diabetes mellitus (DM) care, provides an index of a patient’s average blood glucose (BG) level over the prior three months. It is recognized as the primary outcome measure for evaluating glycemic control and is a strong predictor of diabetes complications and related mortality risk [2]. The American Diabetes Association (ADA) and European Association for the Study of Diabetes (EASD) recommends measurement of HbA1c every three to six months depending on the stability of glycemic control [3]. Thus, as the clinical gold standard measure of glycemic control, this test provides important feedback to healthcare providers and patients.

In recent years, public health campaigns have promoted awareness in patients with diabetes regarding their target and actual HbA1c values, blood pressure and cholesterol levels, so as to be proactive in discussing these with their healthcare providers. However, data suggests that many patient populations worldwide have some, but not adequate knowledge of the HbA1c test. For example, a recent international survey of 661 patients in online health communities in the United States, Mexico, Canada and Europe reported variable awareness of their last HbA1c (42%-89%) and A1C target (26%-70%) [4]. In a study from India, greater than 70% of the subjects had awareness about the HbA1c test, but only 33% remembered their last HbA1c value and less than half of those who knew HbA1c test also knew their glycemic target [5]. Previously, researchers evaluated patient HbA1c awareness by surveying 686 U.S. adults with type 2 DM (T2DM). Of these, 66% of the respondents did not know their last HbA1c value, and only 25% of respondents accurately reported the value [6]. Poor HbA1c awareness has been demonstrated in multiple other populations [7-9] A prospective clinical case series examining HbA1c awareness in individuals receiving screening for diabetic complications demonstrated understanding of HbA1c definition in only 51% of patients examined for diabetic retinopathy [10]. Improving education of glycemic targets amongst patients may predict achievement of target HbA1c at six months [11].

The HbA1c test and interpretation can be taught both formally and informally. For example, at our endocrinology and diabetes center, each examination room is equipped with a color-coded poster denoting general HbA1c target zones (blue zone HbA1c = “good control”, red zone HbA1c = “improved control needed”), and depicting HbA1c to average glucose correlation [12]. Each patient receives a point-of-care HbA1c at each visit, the results and interpretation of which are provided to each patient. The objective of this study was to assess the knowledge of the HbA1c diabetes marker and glycemic control in an urban patient population.

## Materials and methods

Study design

We surveyed 100 patients with diabetes at the University of Maryland Center for Diabetes and Endocrinology (UMCDE), primarily to assess the knowledge and awareness of HbA1C in our patient population. Prior to survey initiation, we hypothesized that there would be a correlation between HbA1C awareness and better glycemic control in our population. The survey was conducted as a non-randomized, clinical cross-sectional study, along with an Institutional Review Board (IRB)-approved retrospective chart review over a four-month period (September and October 2016, February and March 2017).

Study population

Male and female patients with age ≥ 18 and < 80 years, who were new or established patients seen at UMCDE, willing and able to consent and complete all study procedures, were included. Subjects with any degree of cognitive impairment were excluded.

Data collection

**Figure 1:**
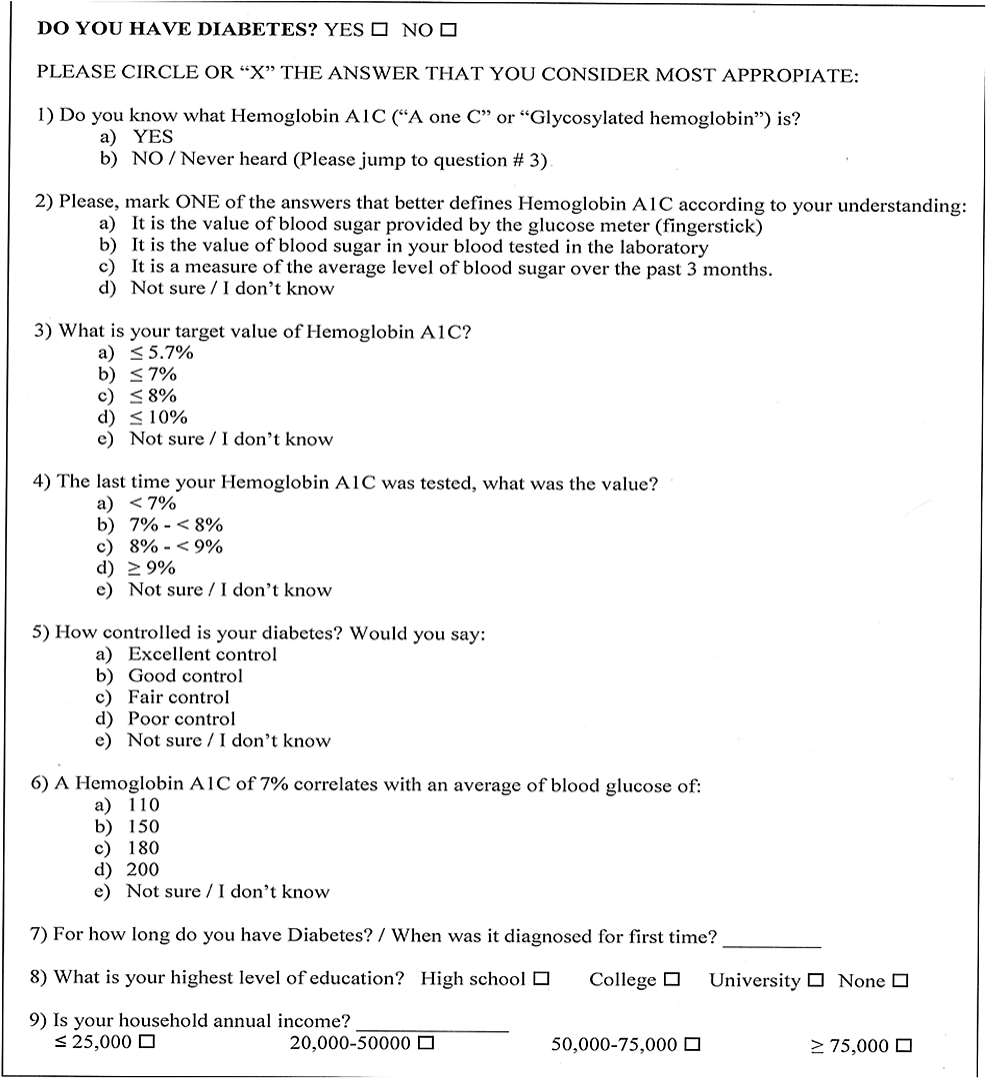
Patient survey

Prior to survey administration, per IRB instruction, we obtained signed consent from patients to conduct the survey, and signed Health Insurance Portability and Accountability Act release for chart review. Subsequently, patients were handed a paper copy of our survey, which included nine, primarily multiple-choice questions (Figure 1).

Subsequent to survey completion, we conducted a chart review to evaluate patient demographics (age, ethnicity, new vs. return patient), body mass index (BMI), type of DM, years since diagnosis of DM, DM regimen, history of hypertension, hyperlipidemia, whether or not patient had received diabetes education by a certified diabetes educator (CDE) and number of sessions received. Baseline demographics are summarized in Table 1.

**Table 1:** Patient demographics HbA1C = hemoglobin A1C, DM = diabetes mellitus, T1DM = type 1 diabetes mellitus, T2DM = type 2 DM, GLP-1 = glucagon-like peptide-1, MDI = multiple daily injection, BMI = body mass index. *Due to the small number of subjects, other races were not included in the statistical analysis. **Due to the small number of subjects, other types of DM were not included in the analysis.

Patient characteristics	HbA1c < 8.0%, n = 51 (%)	HbA1c ≥ 8.0%, n = 48 (%)	P-value
Average age	59.8 ± 11.5	52.1 ± 13.8	0.002
Gender (%)			0.46
Male (45)	25 (49)	20 (41.7)	
Female (55)	26 (51)	28 (58.3)	
Race (%)			0.00097*
White (24.2)	19 (37.3)	5 (10.4)	
African American (72.7)	29 (56.8)	43 (89.6)	
Other (Asian, European) (3.1)	3 (5.9)	0	
Education (%)			0.17
High school/none	26 (51)	31 (64.6)	
College/university	25 (49)	17 (35.4)	
Annual income (%)			0.11
< $25,000	22 (43.1)	19 (39.6)	
$25,000-50,000	10 (19.6)	18 (37.5)	
$50,000-75,000	3 (6)	5 (10.4)	
>$75,000	12 (23.5)	4 (8.3)	
Not reported	4 (7.8)	2 (4.2)	
New to clinic (%)	4 (7.8)	10 (20.8)	0.064
Follow-up visit	47 (92.2)	38 (79.2)	
Type of DM (%)			0.59**
T1DM (11.1)	5 (9.8)	6 (12.5)	
T2DM (85.9)	46 (90.2)	39 (81.2)	
Other (3)	0	3 (6.3)	
Average HbA1C	6.9 ± 0.6%	10.2 ± 1.8%	0.00001
Duration of DM (years)	4.7 ± 8.5	5.6 ± 8.5	0.6
DM regimen (%)			0.0028
No med/orals only	25 (49)	8 (16.7)	
Orals/GLP-1A + insulin	16 (31.4)	23 (47.9)	
MDI	10 (19.6)	17 (35.4)	
Average BMI	32.3 ± 6.2	34.1 ± 10.5	0.3
Hypertension	45 (88)	37 (77)	0.14
Normotensive	6 (12)	11 (23)	
Hyperlipidemia			0.011
Present	47 (92)	35 (73)	
Absent	4 (8)	13 (27)	
Retinopathy			0.049
Present	3 (6)	9 (19)	
Absent	48 (94)	39 (81)	
Neuropathy			1
Present	17 (33)	16 (33)	
Absent	34 (67)	32 (67)	
Nephropathy			0.009
Present	6 (12)	16 (33	
Absent	45 (88)	32 (67)	
Diabetes education			0.11
Yes	33 (65)	38 (79)	
No	18 (35)	10 (21)	
Average no. of sessions	3.88 ± 2.8	3.71 ± 3.5	

Statistical analysis

For data analysis, we divided our patient cohort into those with recent HbA1c < 8.0% (n = 51) and those with HbA1c ≥ 8.0% (n = 49). Current standard of care is to provide individualized HbA1c goals for each patient [13]. For the purpose of this study, we categorized patients according to whether their actual HbA1c was above or below 8%. This demarcation was intended to identify individuals by which most practitioners would agree that an HbA1c > 8% would benefit from improved control and that an HbA1c < 8% would identify most patients with excellent to moderate control. For numerical data, means were calculated. T-test and chi-square tests were used for numerical and categorical data, respectively, to determine the statistical significance of any difference between the groups. In situations where the number of subjects was less than 5, Fisher’s exact test was used.

## Results

A survey of 100 patients was conducted. One patient’s results were excluded from analysis after requesting to be withdrawn from the study.

Patient demographics

Of the 99 patients included in the analysis, 55% of the respondents were female and 45% were male (Table 1) . The patients in the group with HbA1c < 8% were slightly older (mean age 59.8 ± 11.5 years) as compared to the ≥8.0% group (mean age 52.1 ± 13.8, P-value 0.002). In our patient cohort, 72.7% reported race as African American, 24.2% non-Hispanic White, and 3.1% as Asian or other. Information regarding race was obtained from patient's electronic medical record. Mean BMI was similar between the two HbA1c subsets. In terms of level of education, 53% self-reported as high school graduates, 43% reported as college/university graduates, and 4% reported no high school graduation. There was a trend toward increased higher education frequency in the HbA1c < 8.0% subset, but this was not statistically significant. Only 16.2% of our patients reported annual household income greater than $75,000, whereas 41.4% reported annual income less than $25,000. On the basis of income, the two HbA1c cohorts were not significantly different. The majority of patients (86%) were established patients at the practice.

Our study cohort primary consisted of patients with T2DM (85.9%), 11.1% with T1DM and 3% with other types of diabetes. In comparison, the 2015 national data from ADA reports the prevalence of T1DM amongst the DM population to be 4.13% (1.25 million) [14]. The difference between percentage of patients with type 1 versus type 2 diabetes was not statistically different between the two groups. Mean HbA1C prior to survey administration was 8.5 ± 2.1%. There was a 3.3% difference in mean HbA1C between the subset of patients with HbA1C < 8.0% and ≥ 8.0%. In our patient population, the mean duration of DM was 62.4 months; however, the duration of DM was slightly longer (10 months) in the HbA1c ≥ 8.0% subset. Over half of our patients (66.7%) required daily insulin (basal and/or multi-dose insulin), out of which, 61% had their last HbA1C ≥ 8.0%. Our patient cohort had a high prevalence of comorbidities: 83% hypertension, 83% hyperlipidemia, 34% neuropathy, 23% nephropathy, 12% retinopathy. The presence of retinopathy and nephropathy was significantly higher in the subset of patients with HbA1C ≥ 8.0% (P-values 0.049 and 0.009, respectively), while hyperlipidemia was more prevalent in those with HbA1C < 8% (P-value 0.011).

Survey responses

The majority (71%) of the survey respondents correctly defined HbA1c as a measure of average BG level over the preceding three months. There was no statistically significant difference between the two groups in terms of the knowledge of the correct HbA1c definition (Table 2). Despite awareness of HbA1c definition, the cohort was not as knowledgeable regarding HbA1c targets or the correct BG correlate for an HbA1c of 7.0%. Only 15% of our patient cohort knew an HbA1C of 7.0% correlated with BG 150 mg/dL, while 34% thought the BG correlate was 110 mg/dL. The cohort survey responses are summarized in Table 2. Of the patients with last HbA1C < 8.0%, 21.6% reported an aggressive perceived HbA1c target ≤ 5.7%, with similar responses reported by the HbA1c ≥ 8.0% subset. Using a scale, from excellent-to-good-to-fair or poor, 83.3% of the patients with HbA1c ≥ 8.0% reported poor control, but 41.1% patients with HbA1c < 8.0% also reported having fair/poor glycemic control.

**Table 2:** Survey responses HbA1C = hemoglobin A1C, POC = point of care, BG = blood glucose, DM = diabetes mellitus *Due to 0 number of subjects in the group with perceived HbA1c <10%, this group was excluded from analysis. Those who answered “not sure” were also excluded from the analysis. **Those who answered “not sure” were also excluded from the analysis.

	Total	HbA1c < 8.0% (n = 51)	HbA1c ≥ 8.0% (n = 48)	P-value
Knew HbA1C definition (%)	83	41 (80.3)	42 (87.5)	0.33
HbA1C definition (%)				
Reflects POC glucose	6	2 (3.9)	4 (8.3)	0.39
Reflects laboratory BG	7	3 (5.9)	4 (8.3)	
Reflects 3-month BG pattern	70	35 (68.6)	35 (73)	
Not sure/did not answer	16	11 (21.6)	5 (10.4)	
Perceived HbA1c target (%)				
≤5.7%	20	11 (21.6)	9 (18.8)	0.54*
<7.0%	58	30 (58.8)	28 (58.3)	
<8.0%	4	1 (1.9)	3 (6.2)	
<10.0%	0	0	0	
Not sure	17	9 (17.7)	8 (16.7)	
Perceived last HbA1C (%)				
<7.0%	18	16 (31.4)	2 (4.2)	<0.00001**
7.0% - <8.0%	26	21 (41.2)	5 (10.4)	
>8.0%	35	2 (3.9)	33 (68.7)	
Not sure	20	12 (23.5)	8 (16.7)	
Perceived DM control (%)				
Excellent/good	35	29 (57)	6 (12.5)	0.00001
Fair/poor	61	21 (41.1)	40 (83.3)	
Not sure	3	1 (1.9)	2 (4.2)	
HbA1C 7% BG correlate (%)				
110 mg/dL	34	18 (35.3)	16 (33)	0.97
150 mg/dL	15	7 (13.7)	8 (17)	
180 mg/dL	7	4 (7.8)	3 (6.2)	
200 mg/dL	5	2 (3.9)	3 (6.2)	
Not sure	38	20 (39.2)	18 (37.5)	

Patient perception about HbA1C and DM control

Most of our patients (80%) reported knowing their last HbA1c (Table 3). Of these 79 patients, the perceived HbA1c was congruent with the measured HbA1c for 75% of the patients. In 46% of the total patient cohort, their perceived control was congruent with the actual control. More patients in the group with HbA1c ≥ 8% had their perceived control congruent with the actual control as compared to the group with HbA1c < 8% (P = 0.026). However, of those in whom the glycemic control was not congruent with the perceived, 89.5% of those with HbA1c ≥ 8% perceived their control to be better than it actually was. In contrast, in the group which HbA1c < 8%, 96.9% perceived their controlled worse than it actually was (P < 0.00001).

**Table 3:** Patient perception about HbA1c and DM control HbA1C = hemoglobin A1C, DM = diabetes mellitus

	Total (n = 99)	HbA1c < 8.0% (n = 51)	HbA1c ≥ 8.0% (n = 48)	P-value
Congruence of HbA1c with perception (%)				
Did not know last HbA1c	20	12 (23.5)	8 (16.6)	-
Perceived HbA1c congruent with measured HbA1c				
Yes	59	32 (62.7)	27 (56.3)	0.14
No	20	7 (13.8)	13 (27.1)	
HbA1c:				
Perceived < measured	11	2 (28.6)	9 (69.2)	0.08
Perceived > measured	9	5 (71.4)	4 (30.8)	
Congruence of DM control with perception (%)				
Did not report control	3	1 (2)	2 (4.2)	-
Perceived control congruent with actual control				
Yes	45	18 (35.3)	27 (56.2)	0.026
No	51	32 (62.7)	19 (39.6)	
DM control				
Perceived better than actual	18	1 (3.1)	17 (89.5)	<0.00001
Perceived worse than actual	33	31 (96.9)	2 (10.5)	

## Discussion

Our study provides a cross-sectional assessment of the knowledge and perception of glycemic targets in an urban population, at an academic diabetes center. The HbA1c test is both a diagnostic test used to make management decisions and a narrative tool to place an individual’s glycemic control in context of his or her disease. Because the HbA1c test is such a fundamental tool for the practitioner, understanding how the patient experiences this result is critical to a transparent practitioner/patient interaction.

Compared to previous studies, our population was more likely to know the results of their HbA1c test [6,10]. For example, in comparison to the U.S. study [6], in which 66% of the respondents reported not knowing their last HbA1C and only 25% reporting the value accurately, 80% of our patients reported knowing their last HbA1C and 60% had reported it accurately. However, there was no statistically significant difference between the two HbA1C subgroups to show if this was related to better glycemic control. Our results are encouraging, as the first step in the conversation about the usefulness of this test is for the patient to know the result. This is likely the benefit of point-of-care testing provided in this practice. The turn-around time for the result is generally less than six minutes affording the practitioner real-time results to review. The practitioners were also able to correlate the HbA1c with the last three months of BG control provided by the patient. This may reflect that most visits are on a three-month appointment cycle giving natural reinforcement of the HbA1c time frame. Additionally, our cohort correctly perceived a goal HbA1c target of <7%, which is generally consistent with ADA/EASD goals for most patients. While a group of individuals did perceive an even stricter HbA1c target (≤5.7%), this was consistent across groups and may reflect patients’ idealization of achieving “normal” glucose control.

Unfortunately, patients were unable to recognize the relationship between the HbA1c value and average finger stick glucose levels. Improved glycemic control, as reflected by the last HbA1c measurement, did not improve the understanding of the BG-HbA1c correlate (Table 2). Most patients perceived that an HbA1c test of 7% was associated with an average BG of 110 mg/dL rather than 150 mg/dL. It reinforces that patients may not understand how to use their home glucose monitoring to assess their overall control in an accurate manner. This is an opportunity for further education. It is a nuance of the HbA1c test that can give patients more autonomy and ownership of their disease. By understanding this relationship, they can anticipate their HbA1c and glycemic control before the visit.

Our patient population was primarily African American, and this was reflected in both HbA1c groups. When the groups are compared, the HbA1c ≥ 8.0% group was disproportionately more African American (90%, P = 0.000097). The existence of racial and ethnic variations in the HbA1c must be taken into consideration [15-17]. However, it is also thought that race may only partially explain this variation. Other socioeconomic factors that pose a barrier to care in certain ethnic groups should be studied in greater detail [18,19]. Further ongoing research comparing the racial difference in finger stick glucose levels and perhaps continuous glucose monitoring would be valuable. There was no difference in the duration of diabetes, whether they were new or established patients of the clinic or whether they had received diabetic education. This is in contrast to previous studies [8,20,21]. This does not appear to reflect the impact of socioeconomic disparities on the glycemic control and health outcomes in this urban population, as there was no difference in the education level or annual income between these groups. This finding is an opportunity for further research to understand this difference.

Interestingly, the majority of patients in the HbA1c ≥ 8.0% group perceived their glycemic control to be fair or poor, which is generally accurate based on their HbA1c. In addition, this group’s perception of glucose control was more likely to be congruent with the actual control compared to the HbA1c < 8% group. In general, the HbA1c ≥ 8.0% group patients are more likely to recognize that they are uncontrolled that is an opportunity to engage with the patients, reinforce goals and provide support. Additionally, individuals in the HbA1C ≥ 8.0% group, whose perception of control is not accurate, are more likely than the <8% group to perceive their control to be better than it actually was. It is possible that poor control portends to improved control perception, as prior studies have demonstrated that this cohort feels physically better at higher BG levels and requires a higher hyperglycemic threshold prior to experiencing hyperglycemic symptoms [22]. This is a critical group to consider the mode in which a practitioner relays information. For some individuals, this may be auditory or visual. In this uncontrolled group whose control is poor and perception is inaccurate, it may be important to ask the patients the manner in which they learn information and to consider using teach-back methods to ascertain comprehension of the HbA1c result [23].

Interestingly, the group with HbA1c < 8% whose perceived DM control was not congruent with their actual DM control were more likely to perceive their DM control as worse than it actually was. This may reflect patient’s modesty, vigilance or an inaccurate reflection on expectations. Recent guidelines recommend individualized targets for glycemic control, highlighting the risks of intensive glycemic targets for different groups of individuals with DM. In this group, a discussion may include the reason(s) they cannot attain stricter control.

This study was limited to a single academic clinic with predominantly African Americans. Our results and conclusions may not apply to other populations with diabetes. Some of our survey results did fit clinical intuitions but did not achieve statistical significance. This might have improved by increasing the sample size. Assessing patient perception of teaching methods, tools and techniques may be areas of future research.

## Conclusions

In conclusion, patients’ education regarding their HbA1c and their goal HbA1c level continues to be a cornerstone of the diabetes treatment plan. Although most providers would expect that the HbA1c result and the discussion regarding this important clinical test are translated to patients, there remains a disconnect for patients between their HbA1c and blood glucose monitoring.
